# Seeking pleasant touch: neural correlates of behavioral preferences for skin stroking

**DOI:** 10.3389/fnbeh.2015.00008

**Published:** 2015-02-05

**Authors:** Irene Perini, Håkan Olausson, India Morrison

**Affiliations:** ^1^Department of Clinical and Experimental Medicine, Linköping UniversityLinköping, Sweden; ^2^Institute of Neuroscience and Physiology, University of GothenburgGothenburg, Sweden; ^3^Department of Clinical Neurophysiology, Linköping University HospitalLinköping, Sweden

**Keywords:** fMRI, CT afferents, affective touch, seeking behavior, interoception

## Abstract

Affective touch is a dynamic process. In this fMRI study we investigated affective touch by exploring its effects on overt behavior. Arm and palm skin were stroked with a soft brush at five different velocities (0.3, 1, 10, 3, and 30 cm s^−1^), using a novel feedback-based paradigm. Following stimulation in each trial, participants actively chose whether the caress they would receive in the next trial would be the same speed (“repeat”) or different (“change”). Since preferred stroking speeds should be sought with greater frequency than non-preferred speeds, this paradigm provided a measure of such preferences in the form of active choices. The stimulation velocities were implemented with respect to the differential subjective pleasantness ratings they elicit in healthy subjects, with intermediate velocities (1, 10, and 3 cm s^−1^) considered more pleasant than very slow or very fast ones. Such pleasantness ratings linearly correlate with changes in mean firing rates of unmyelinated low-threshold C-tactile (CT) afferent nerves in the skin. Here, gentle, dynamic stimulation optimal for activating CT-afferents not only affected behavioral choices, but engaged brain regions involved in reward-related behavior and decision-making. This was the case for both hairy skin of the arm, where CTs are abundant, and glabrous skin of the palm, where CTs are absent. These findings provide insights on central and behavioral mechanisms underlying the perception of affective touch, and indicate that seeking affective touch involves value-based neural processing that is ultimately reflected in behavioral preferences.

## Introduction

Affective touch is an emotional form of social interaction, and the only one that relies on bodily contact. Its importance for social connection and well-being is essential from infancy onward (Hertenstein and Campos, 2001; Muir, 2002; Hertenstein et al., 2006; Ardiel and Rankin, 2010; Morrison et al., 2010; Fairhurst et al., 2014). However, little is known about the neural mechanisms by which affective touch affects behavior in social interactions. In this study we ask whether affective touch informs overt behavioral choices, and if so, what are the neural correlates underlying the evaluations and decisions driving such behavior.

The hedonics of human touch and associated behavior are complex. On the psychological and behavioral levels, interpersonal touch interactions can be driven by different motivations—for example, to comfort or be comforted, to initiate intimacy, or to communicate emotions. On the cortical level, motivational and hedonic components of touch may interact, making it difficult to disentangle the hedonic appreciation of social touch from the motivational impetus to seek it. Finally, on the level of subjective experience, hedonic representations originating in different parts of tactile, motivational, reward, and other systems may give rise to unitary percepts, which do not necessarily decompose readily into distinct subcomponents in the lab.

What are the candidate neural pathways that may influence the hedonic evaluation of touch and, ultimately, overt behavioral choices? Recent evidence suggests that unmyelinated C-tactile (CT) afferent nerve fibers in human skin convey signals related to the hedonic value of a soft caress (Vallbo et al., 1999; Olausson et al., 2002; Wessberg et al., 2003; Löken et al., 2009; Morrison, 2012). CTs are found only in hairy (e.g., arm) but not glabrous (e.g., palm) skin. They show a unique tuning to a narrow range of caressing speeds of about 1–10 cm s^−1^, peaking at about 3 cm s^−1^ (Löken et al., 2009). Crucially, mean CT firing frequency during caress stimulation correlates with pleasantness ratings (Löken et al., 2009). CT firing frequency also increases during skin-temperature stroking (32°C), compared to cooler and warmer temperatures, further suggesting a socially-relevant function (Ackerley et al., 2014). Further, selective activation of a mouse homolog of low-threshold, unmyelinated CT afferents can alter a mouse’s behavioral preferences (Vrontou et al., 2013).

With this candidate pathway as a starting point, we therefore hypothesized that stroking participants’ skin at different speeds (0.3, 1, 10, 3, and 30 cm s^−1^) and on different skin types (hairy and glabrous) would differentially affect their hedonic evaluation and preferences, and give rise to different patterns of behavioral and neural activation. These speed and location conditions were therefore incorporated in a novel paradigm designed to characterize hedonic evaluation in terms of its impact on behavioral choices for both hairy and glabrous skin. The feedback-based paradigm allowed participants to choose the speed of gentle brushing stimulation they received. In each trial, the subject received brush strokes on the forearm (hairy skin) or palm (glabrous skin) at one of the five different speeds. After each trial, participants indicated by button-press whether they would rather receive the same stroking speed again (“repeat”) or change to another one, randomly selected by the computer (“change”). This was designed to approximate everyday interpersonal touch behavior, in which individuals decide to seek a specific touch stimulus, perhaps based on experience or past evaluations.

This paradigm allowed us to capture four main aspects of dynamic and affective tactile stimulation. First, we explored general activation during dynamic touch stroking, regardless of stimulation speed, skin surface, and behavioral outcome. Second, we explored the evaluation of this stimulation with respect to a behavioral decision, by examining all activation following stroking but preceding an active behavioral choice. To discover signal increases for repeat vs. change choices, we compared these conditions within the evaluation period. Within this evaluation period we also targeted cerebral activation increases to preferred touch stimulation speeds. Finally, we investigated the neural correlates of specific behavioral preferences by examining preferred vs. non-preferred speeds on arm and palm. In this way we were able to explore aspects of affective touch stimulation not only with respect to stimulus processing, but also to how evaluation of such stimulation influences hedonic preferences and overt behavioral choices.

## Methods

### Participants

Eighteen neurologically healthy subjects, recruited from the University of Gothenburg participated in the study (age 20–32, 9 males). The procedures were approved by the ethics committee of the University of Gothenburg, in accordance with the Declaration of Helsinki. Participants gave informed consent and were compensated at 200 Swedish crowns (22 Euro) per session.

### Stimuli and design

Stimuli consisted of single brush strokes over 5 cm of left forearm or palm skin using a soft 70 mm-wide goat hair artist’s brush, brushing in a distal to proximal direction. Brush strokes were delivered manually at 5 different velocities: 0.3 cm s^−1^, 1 cm s^−1^, 3 cm s^−1^, 10 cm s^−1^, and 30 cm s^−1^. Presentation of the initial velocity was random, and after the first trial subjects could choose the velocity presented in the next trial by pressing one of two buttons with their right hand, indicating whether to repeat the previous velocity (“repeat”) or to change to a new velocity randomly selected by the computer program (“change”).

**Figure 1:**
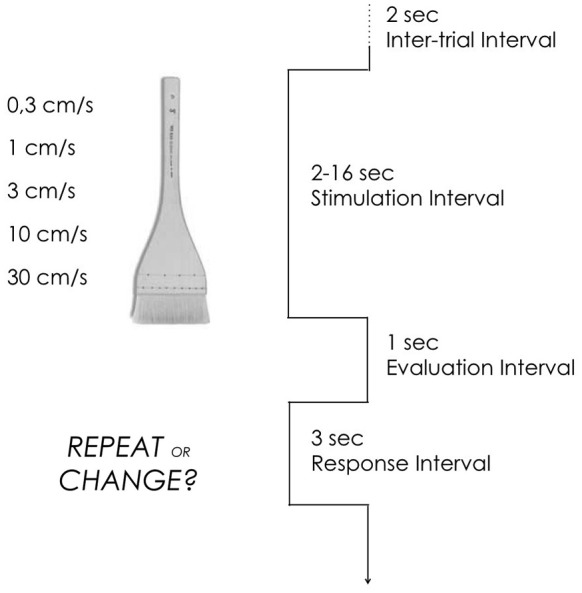
**Design**. Each trial began with a 2 s inter-trial interval during which subjects fixated their gaze on a dot presented centrally on the screen. There followed a 2–16 s stimulation interval, depending on the velocity delivered. Participants received single brush strokes on the arm at different velocities. Stimulation consisted of single brush strokes delivered manually on 5 cm of the arm or the palm. A 1 s interval occurred between stimulation and the onset of the response cue. The response cue consisted of the sentence “Repeat or change?” remaining on the screen for 3 s as the subjects pressed the button indicating their choice.

Each trial began with a 2 s inter-trial interval during which subjects fixated their gaze on a dot presented centrally on the screen. There followed a stimulation interval lasting between 2 and 16 s, depending on the velocity delivered. A 1 s interval occurred between stimulation and the onset of the response cue. The response cue consisted of the sentence “Repeat or Change?” remaining on the screen for 3 s as the subjects pressed the button indicating their choice (Figure 1).

The experimental design was a 2 × 5 factorial with the factors location (arm, palm) and velocity (0.3, 1, 3, 10, 30 cm s^−1^). Data were collected in two runs during one scanning session, with arm and palm stimuli presented in separate runs to avoid movement artifacts associated with turning the arm to and from a pronated position. Run order was counterbalanced across subjects. Importantly, since the design was feedback-based, the number of trials and volumes per run varied between subjects. To counteract “shift” biases while ensuring a minimum number of stimulations per velocity for all participants, each velocity was presented a baseline minimum of 6 times over the whole run. To counteract “stay” biases, the program automatically changed to a different randomly selected velocity after two consecutive repeats. The subjects were aware that they would receive a maximum of three trials of the same velocity in a row.

Response mapping on the button box was counterbalanced across subjects to avoid spatial confounds (i.e., “repeat” or “change” with index or middle finger). The experimenter (I.P.) was trained in the delivery of the stimuli, and during the experiment was guided by a visual meter with a moving stripe representing the velocity and distance in each trial. Visual cues to the participant were projected onto a screen positioned near the participants’ feet, visible through mirrors affixed to the head coil. Occluders were placed on the mirror to ensure that participants’ field of view was limited to the screen and did not include the stimulated limb or the experimenter. Each run began and ended with a fixation period of 45 s to allow a return to baseline.

### FMRI data acquisition

A 1.5 T Philips Intera magnetic resonance imaging (MRI) scanner with a SENSE head coil was used. For functional imaging, a single-shot echo-planar imaging sequence was used (T2*-weighted, gradient echo sequence, repetition time (TR) = 3000, echo time = 50 ms, flip angle = 90º, field-of-view (FOV) 230 mm). The scanned area included 30 axial slices, 5 mm thick, with no gap, at 64 × 64 voxel in-plane resolution, which covered the whole cerebral cortex and the cerebellum. To minimize head movement, participants’ heads were stabilized with a vacuum hood filled with polystyrene balls (Vacuform Hood, Cambridge Research Systems, Cambridge, UK).

### Behavioral data analysis

Behavioral data were analyzed using Statistical Package for the Social Sciences (SPSS Inc., Chicago, IL, USA). To obtain a measure of preference per velocity, the mean ratios of the number of repeats for all active choices (total number of repeats plus total number of changes) for each velocity were converted to percentage values. A chance cutoff of 45% (*p* < 0.05) was imposed based on the binomial distribution of the responses. Therefore velocities for which the subjects showed an above-chance mean of >45% selection of “repeat” over “change” responses were taken to indicate a positive preference for brush stroking stimulation at that velocity; those <45% were taken to indicate no preference or negative preference.

To investigate effects of stroking velocity and location, percentage values were then submitted into a 2 × 5 factorial analysis of variance (ANOVA) with factors location (arm, palm) and velocity (0.3, 1, 3, 10, 30 cm s^−1^). Further analysis was conducted to assess the curve of the percentage value pattern across velocities to and address potential similarities with the pleasantness’ rating pattern across velocities (Löken et al., 2009). To investigate this aspect, regression analyses were performed on arm and palm percentage rating values separately. The factor velocity was used as independent variable, logarithm-transformed and entered as linear and quadratic terms in a regression model. In addition, the shapes of the resulting regressions for arm and palm were statistically compared by performing a linear mixed model in which both arm and palm ratings were taken into account.

### FMRI data analysis

Preprocessing and statistical analysis of MRI data were performed using BrainVoyager QX (Brain Innovation, Maastricht, The Netherlands) and exploratory ROIs analysis (Poldrack, 2007) was performed with SPSS. Three dummy volumes were acquired before each scan in order to reduce possible effects of T1 saturation. Functional data were motion corrected and low-frequency drifts were removed with a temporal high-pass filter (0.006 Hz). Spatial smoothing was applied with a 6 mm full width at half-maximum filter. Functional data were manually co-registered with 3-dimensional (3D) anatomical T1 scans (1 × 1.58 × 1.58 mm resolution resampled to 1 × 1 × 1 mm), on the basis of anatomical landmarks for each individual. The 3D anatomical scans were transformed into Talairach space (Talairach and Tournoux, 1988), and the parameters for this transformation were subsequently applied to the coregistered functional data.

Six out of 36 runs from a total of six participants were discarded due to excessive head-motion during the experiment (>2 mm). For each participant, three different general linear models (GLMs) were created for each of the 2 runs: “Stimulation interval” capturing activation during the brush stroking on the skin; “Evaluation interval” capturing post-stimulus processing; skin stimulation and “Preference ratio” capturing behavioral preference across velocities for arm and palm.

### GLM 1: stimulation interval

Two predictors modeled the intervals corresponding to the tactile stimulation period in each trial. One predictor (“repeat”) included all trials in which the participant selected “repeat.” The other predictor (“change”) included all trials in which the participant selected “change”. To investigate BOLD signal changes following tactile stimulation, a whole-brain contrast compared all stimulation intervals vs. a fixation baseline (all inter-trial intervals) for both arm and palm. Repeat vs. baseline and change vs. baseline contrasts were also used to address potential differences between arm and palm tactile processing. Whole-brain random effects contrasts were corrected for multiple comparisons using BrainVoyager’s cluster threshold estimator plug-in, which uses a Monte Carlo simulation procedure (1,000 iterations) to establish the critical cluster size threshold corresponding to a family-wise alpha of 0.05 corrected for the whole brain (Forman et al., 1995; Goebel et al., 2006).

### GLM 2: evaluation interval

In this model, two predictors modeled the 1 s interval after tactile stimulation and before button response, during which the subject was preparing to select “repeat” or “change”. This interval captured evaluative processing that is geared towards an overt decision. One predictor for this interval (“repeat”) included all trials in which the participant chose “repeat”. The other predictor for this interval (“change”) included all trials in which the participant chose “change”. Whole brain contrasts between repeat and fixation baseline and a contrast between repeat and change were also performed. Whole-brain random effects contrasts were corrected for multiple comparisons using BrainVoyager’s cluster threshold estimator plug-in, corresponding to a family-wise alpha of 0.05 corrected for the whole brain.

### GLM 3: preference ratio

To reveal which areas represented the behavioral preferences across all five velocities, thus likely reflecting any differential weighting across velocities, a whole-brain search was conducted based on the participants’ individual behavioral ratios of repeat-to-change choices (see Section Behavioral data analysis above). As in the “Evaluation” GLM, each predictor modeled the 1 s interval between stimulus and choice. However in this model, ten predictors were created for each of the 10 conditions (0.3, 1, 3, 10, and 30 cm s^−1^ for arm; 0.3, 1, 3, 10, and 30 cm s^−1^ for palm). Because the number of trials per condition was variable across subjects due to the feedback-based nature of the design, a baseline of the first 6 trials per condition was used to define the predictors for the whole-brain, group average analysis. Using the first trials for each velocity also avoids potential confounds associated with repetition of the same trial type (i.e., none followed a “repeat” choice). Whole-brain random effect contrasts were performed at an uncorrected threshold of *p* < 0.005. We performed a whole-brain search for areas showing higher hemodynamic responses following velocities in which the proportion of “repeats” to “changes” was above a “preference” cutoff, based on the group’s binomial distribution of ”repeat” choices, in which chance was at 45% with a *p* of 0.05; see Section Behavioral data analysis above). Velocities with above-chance percentages were weighted positively and those below chance weighted negatively. Based on the behavioral responses we used the contrast [(1 + 3 + 10) cm s^−1^ > (0.3 + 30) cm s^−1^] for the arm trials and [(3 cm s^−1^ ) > (0.3 + 1 + 10 + 30) cm s^−1^] for the palm trials.

## Results

### Behavior

#### Hedonic preference for touch depends on the speed of stroking

**Figure 2:**
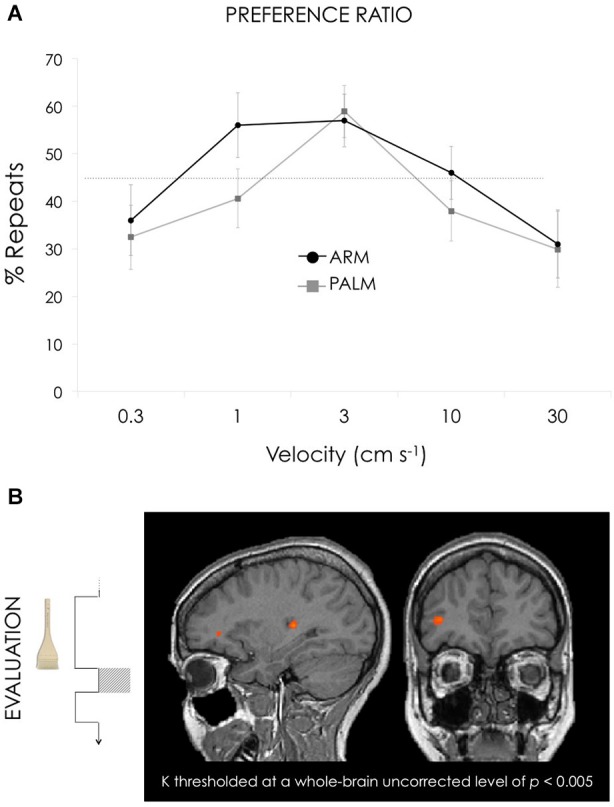
**(A)** The two graphs represent the percentage of the ratio of repeats on overall choices for arm (black) and palm (gray). For the arm, ratio values exceeded chance (45%) for 1, 3, and 10 cm s^−1^ whereas for the palm, the repeat percentages exceeding chance was only for 3 cm s^−1^. Both curves were significantly best described by a negative quadratic term. **(B)** Activation maps for GLM3: Preference ratio, reflecting differential weighting across velocities. To represent the behavioral ratio the following contrast was used: [(1 cm s^−1^ + 3 cm s^−1^ + 10 cm s^−1^) > (0.3 cm s^−1^ + 30 cm s^−1^)] for the arm trials vs. [(3 cm s^−1^) > (0.3 cm s^−1^ + 1 cm s^−1^ + 10 cm s^−1^ + 30 cm s^−1^)] for the palm. This contrast reveals clusters with higher responses for 1, 3, and 10 compared to 0.3 and 30 s^−1^ in the arm conditions; and 3 compared to 0.3, 1, 10 and 30 s^−1^ in the palm conditions. Both arm and palm runs were included in the general linear model. Results revealed activation in right dlPFC (35, 39, 6) and right posterior insula (29, −26, 9). All contrasts thresholded at a whole-brain uncorrected level of *p* < 0.005. Talairach coordinates, radiological convention (left is right).

The total number of trials varied from 36 to 83 for the different subjects. For the arm, the binomial distribution of responses (i.e., mean repeat vs. total choice percentages) exceeded chance (45%) for 1, 3, and 10 cm s^−1^ (Figure 2). For the palm, the repeat percentages exceeding chance was only for 3 cm s^−1^. The percentage values for each velocity for arm were (from slowest to fastest velocities, mean ± SD): 36.1 ± 31.4, 56.1 ± 29.1, 57.4 ± 23.5, 45.9 ± 23.7, 30.7 ± 30.2. For palm were (from slowest to fastest velocities, mean ± SD): 32.4 ± 28.7, 40.6 ± 26.3, 58.8 ± 23.3, 37.9 ± 26.9, 29.8 ± 34.1.

The mean repeat vs. total choice percentages were submitted to a 2 × 5 repeated-measures ANOVA with 2 within-subject factors: location (arm or palm) and velocity (0.3, 1, 3, 10, or 30 cm s^−1^). A significant main effect of velocity was seen, *F*_(2.23,38.06)_ = 5.482, *p* = 0.006 (Greenhouse-Geisser). A negative quadratic term in the regression provided a significantly better fit compared to a linear term both for arm *p* = 0.001 and palm *p* = 0.005 repeat-change ratio data.

### FMRI

#### Stimulation interval: tactile stimulation activates somatosensory areas

**Figure 3:**
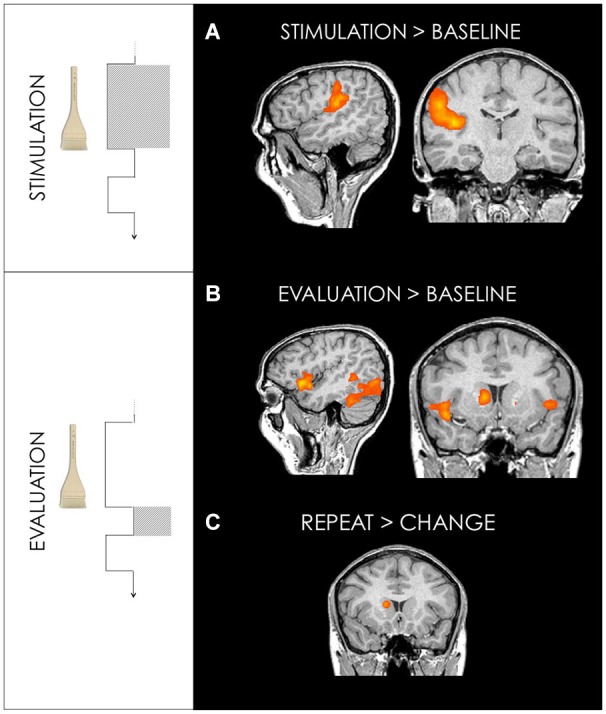
**(A)** Activation maps for GLM1: Stimulation. Brush stroking stimulation revealed activations in somatosensory areas. Right primary somatosensory cortex (29, −38, 54) and secondary somatosensory cortex/posterior insula (38, −14, 12) for stimulation on both arm and palm are shown. **(B)** Activation maps for GLM2: Evaluation Stimulation (Table 2). This interval reflects the 1 s following the stimulation before choice. Bilateral anterior insula (44, 19, −3 and −43, 19, −3) and primary visual cortex (11, −80, 6) are shown. **(C)** The repeat vs. change contrast, revealed activation in the right caudate (14, 13, 15). Random effect contrasts were performed at a corrected threshold of *p* < 0.001. Talairach coordinates, radiological convention (left is right).

Tactile stimulation vs. fixation baseline showed activation in right (contralateral to the stimulated side) primary and secondary somatosensory cortices (SI and SII) and posterior insula (PI) (Figure 3, Table 1).

**Table 1:** **Activation during “stimulation interval”**.

Brain region/contrast	Peak coordinates (Talairach)	Maximum *t*-score	Cluster size (mm^3^)
*All stimulation vs. baseline*			
Right SII/PI	38, −14, 12	8.82	8287
Right MI	53, −2, 33	6.07	446
Right SI	29, −38, 54	6.08	259
*Repeat vs. change*			
Left dorsolateral PFC (BA9)	−1, 40, 33	5.72	150
*Repeat vs. baseline (arm only)*			
Right PI	41, −20, 15	5.86	923
*Repeat vs. baseline (palm only)*			
Right SI	56, −20, 36	6.83	944
Right SII/PI	47, −23, 21	5.61	2919

All contrasts thresholded at p < 0.001, cluster-size corrected at p < 0.05. (SI = Primary Somatosensory, SII = Secondary Somatosensory, PI = Posterior Insula, MI = Primary Motor, PFC = Prefrontal Cortex). Ta

**Table 2:** **Activation for “evaluation interval”**.

Brain region/contrast	Peak coordinates (Talairach)	Maximum *t*-score	Cluster size (mm^3^)
*All stimulation vs. baseline*			
Right VI (BA17)	11, −80, 6	7.91	15,643
Right AI	44, 19, −3	7.41	1883
Left AI	−43, 19, −3	5.54	1417
Left dorsolateral PFC	−19, 46, 24	7.26	934
Left rostrolateral PFC (BA10)	−10, 46, 18	5.28	222
Left rostral PFC (BA10)	32, 58, 12	4.84	442
Right ITG	44, −44, 0	4.77	130
Right thalamus	14, −20, −3	7.24	863
Left thalamus	−19, −26, −3	6.79	1379
Right cerebellum	23, −59, −18	9.98	31,606
Left cerebellum	−25, −56, −21	9.24	19,987
Striatum (Right caudate)	14, −5, 18	9.64	5204
Striatum (Left caudate)	−22, −5, 6	7.58	3137
Left pons	−1, −23, −27	7.40	1266
*Repeat vs. change*			
Left dorsolateral PFC	−37, 37, 36	4.99	347
Right caudate	14, 13, 15	5.34	162
Precentral gyrus (BA4)	53, −5, 45	4.46	218

*All contrasts thresholded at *p* < 0.001, cluster-size corrected at *p* < 0.05. (PFC = Prefrontal Cortex). (VI = Primary Visual, AI = Anterior Insula, PFC = Prefrontal Cortex, ITG = Inferior Temporal Gyrus)*.

**Figure 4:**
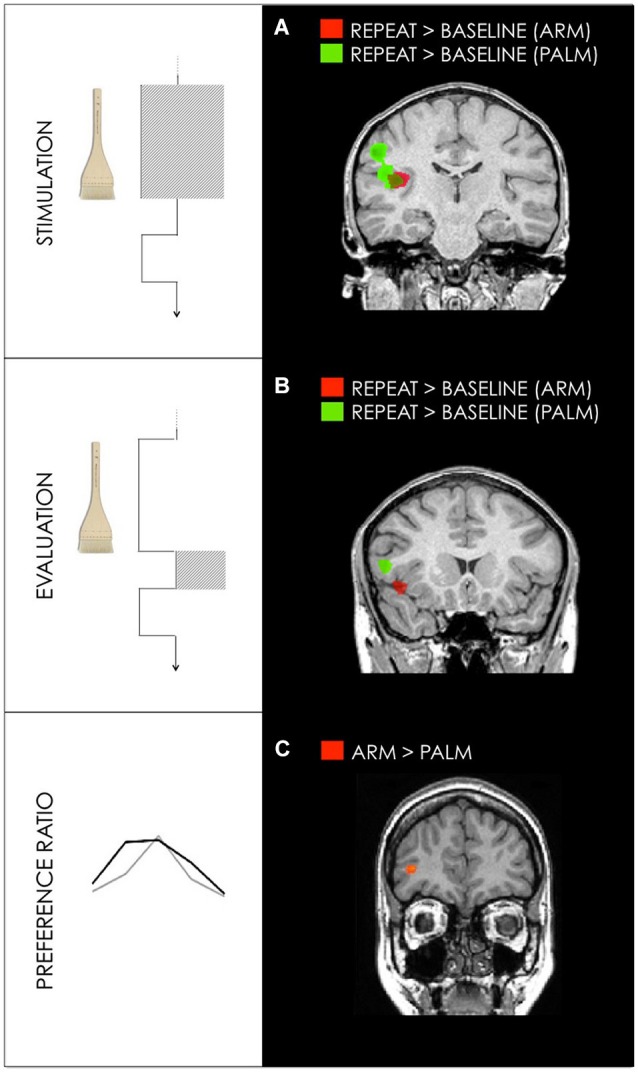
**(A)** Activation maps for GLM1: Stimulation Differences between hedonic stroking vs. fixation for arm (red) and palm (green) during stimulation interval. **(B)** Activation maps for GLM2: Evaluation. Differences between hedonic stroking vs. fixation for arm (red) and palm (green) during the evaluation interval. **(C)** Activation maps for GLM3: Preference ration. Differences between arm and palm for the GLM3 revealed activation in right dlPFC (35, 40, 3). Random effect contrasts were performed at a corrected threshold of *p* < 0.001. Talairach coordinates, radiological convention (left is right).

For all trials in which subjects chose to repeat the stimulation in arm conditions, repeated trials vs. fixation baseline revealed activation in contralateral PI. All repeated trials in palm conditions activated contralateral primary and bilateral secondary somatosensory cortices (Figure 4, Table 1).

#### Evaluation interval: touch evaluation activates interoceptive and reward-related areas

The 1 s interval following stimulation and preceding subjects’ choice of “repeat” or “change”, compared to fixation baseline, revealed activation in bilateral anterior insula, prefrontal cortex, occipital cortex, cerebellum, thalamus and striatum. All ”repeat” vs. “change” trials revealed activity in the head of the caudate, the dorsolateral prefrontal cortex (dlPFC) and precentral gyrus (Figure 3, Table 2).

#### Preference ratio: dlPFC and PI code preferred velocities

Comparing the “above-chance” (preference) to “below-chance” (no preference) velocities during the 1 s post-stimulation “Evaluation” interval preceding button-press revealed peak activations in right dlPFC and right PI (Figure 2, Table 3). A 2 × 5 ANOVA with 2 within-subject factors (location and velocity) revealed a significant main effect of location in dlPFC, *F*_(1,17)_ = 29.312, *p* < 0.001, with higher values for arm (mean beta 0.737 (SE = 0.324)) than palm (mean beta −0.990 (SE = 0.315)).

**Table 3:** **Activation for “preference ratio” contrast, [(1 cm/s + 3 cm/s + 10 cm/s) > (0.3 cm/s + 30 cm/s)] for the arm trials and [(3 cm/s) > (0.3 cm/s + 1 cm/s + 10 cm/s + 30 cm/s)] for the palm trials**.

Brain region/contrast	Peak coordinates (Talairach)	Maximum *t*-score	Cluster size (mm^3^)
*Preference ratio*			
Right PI	29, −26, 9	4.96	113
Right dorsolateral PFC	35, 39, 6	4.38	136
*Arm vs. palm*			
Right dorsolateral PFC	35, 40, 3	4.76	217

*All contrasts thresholded at a whole-brain uncorrected level of *p* < 0.005 (*t* = 3.22). (PI = Posterior Insula, PFC = Prefrontal Cortex)*.

## Discussion

Gentle, dynamic stimulation optimal for activating CT afferents influenced behavioral preferences and engaged brain regions involved in reward-related evaluation and decision-making. This was the case for both hairy skin of the arm, where CTs are abundant, and glabrous skin of the palm, where CTs are absent. The experiment’s novel feedback-based paradigm was designed not only to capture relationships between tactile processing and behavioral preferences, but also to disambiguate the key hedonic components of “liking” and “wanting” (Berridge and Robinson, 2003). “Liking” refers to the estimation of the positive value of a stimulus, whereas “wanting” reflects the impact on behavior following reward-driven changes in motivational states. Together these components guide motivated choices (Berridge and Robinson, 2003) and provide impetus to seeking behavior (Panksepp, 1998).

Previous affective touch experiments have relied on visual analog scales (VASs) to provide subjective measures of hedonic evaluation. Such rating measures correspond to “liking” the stimulus. Choosing to repeat a stimulus more closely taps into “wanting”. Consistent with VAS ratings across stroking speeds for hairy forearm skin (Löken et al., 2009; Morrison et al., 2011a,b), participants chose intermediate, CT-optimal velocities (1–10 cm s^−1^) with above-chance frequency for arm stimulation, but not the very slow or very fast speeds less likely to activate CTs (0.3 and 30 cm s^−1^). This corroborates the previous VAS evaluations, but using an orthogonal measure that does not rely on explicit semantic labeling of the stimulus as “pleasant.” In the palm skin, which lacks CT afferents, only 3 cm s^−1^ stroking was selected with above-chance frequency, suggesting a narrower range of hedonic preference in glabrous skin. The behavioral results supported the hypothesis that the positive valence of affective touch also carries motivational value.

### Stimulation interval

On the level of the brain, any hedonic evaluation of touch is based on processing of its properties during stimulation. Here, all tactile stimulation (both preferred and non-preferred) activated contralateral PI and SII for both arm and palm, compared to a fixation baseline. PI activation is consistent with previous studies of selective CT stimulation (Olausson et al., 2002). Converging evidence indicates that this region is an early cortical target for an afferent pathway including CTs (Olausson et al., 2002; Craig, 2009). This area is associated with somatosensory processing and is highly interconnected with somatosensory networks (Augustine, 1996). Functional and connectivity evidence indicates that sensory information may be integrated in a caudo-rostral fashion within the insula (Kurth et al., 2010; Cerliani et al., 2012). The PI’s contribution to somatosensation may lie in its dense inputs from spinothalamic pathways (Dum et al., 2009), strongly implicated in “interoceptive” representation, as well as its connections with anterior insula (Craig, 2002, 2009; Björnsdotter et al., 2009, 2010; Morrison et al., 2011a).

The engagement of somatosensory cortices, particularly for palm stimulation, is consistent with discriminative encoding, with a predominant contribution from large, fast-conducting Aß tactile afferent pathways (Trulsson et al., 2001; McGlone et al., 2002; Kandel et al., 2012). Most tactile input from Aß afferents in the skin follows a pathway with terminations in somatosensory cortices, associated with high-acuity stimulus discrimination.

PI and somatosensory cortices were activated for both arm and palm stroking. However, preferred arm stroking was limited to PI whereas preferred stroking on the palm also engaged parietal primary and secondary somatosensory areas (Figure 4; see also McGlone et al., 2012 for similar arm-palm differences). Together with the palm-specific activation in SI, this incomplete overlap between arm and palm stroking activation suggests a general bias towards arm (CT + Aβ input) in PI, alongside a bias towards palm (Aβ input) in somatosensory cortices. This difference in bias for arm and palm activation, with arm responses limited to PI, suggests that the PI may be sufficient for coding hedonic touch in hairy skin. The additional engagement of discriminatory areas by palm stroking could reflect a partially distinct contribution to hedonic processing (Pleger et al., 2008; Gazzola et al., 2012; McGlone et al., 2012). It also indicates that different skin types involve different, yet related, processing on the cortical level.

### Evaluation interval

After stimulation and before the button-response cue, there was a 1 s interval during which participants prepared to choose “repeat” or “change.” During this evaluation interval there was strong bilateral activation of anterior insula, regardless of stroking speed or behavioral choice. This supports the idea of anterior insula as a hub of complex interoceptive processing for implementation of appropriate behavior (Craig, 2009). It also highlights the importance of subjective internal states before decisions and related behavioral outcomes (Paulus, 2007; Lovero et al., 2009; Noël et al., 2013). Anterior insula activation was independent of repeat or change choices, yet differentiated between arm and palm stimulation. Specifically, activation for arm-related signal changes was centered in contralateral anterior insula whereas the palm activation was centered more ventrally in inferior frontal gyrus (Figure 4).

The areas showing selective activation for repeats vs. changes were in the precentral gyrus, left dlPFC and the head of the caudate. The caudate is associated with goal-directed behavior and reward expectancy (Kawagoe et al., 1998; Schultz, 2000; Kable and Glimcher, 2007; Lau and Glimcher, 2007; Pleger et al., 2009). It also plays a fundamental role in the preparation of movements that lead to a rewarding outcome (Hollerman et al., 1998) and in behavioral learning (Haruno et al., 2004). Specific “repeat”-related activation in the caudate is consistent with its engagement in reward-related behavior. This activation provides evidence that striatal, reward-related regions participate in the evaluation of a specific tactile stimulus.

### Preference ratio

Participants preferred arm stroking at 1, 3, and 10 cm s^−1^, and palm stroking at 3 cm s^−1^, choosing to repeat rather than change away from these speeds significantly more often. The percentage of repeat to change trials for each stroking speed formed a binomial distribution, and speeds which fell above this distribution’s chance-level likelihood of choosing “repeat” were considered preferred stimuli. All preferred vs. non-preferred speeds regardless of skin type activated PI and dlPFC. Because this contrast was based on a ratio of repeats to changes for each participant, it captured processing in regions that take *both* preferred and non-preferred stimuli into account. The PI and dlPFC activations therefore suggest that these areas are involved in value-based choices reflecting preferences among tactile brush strokes at different stimulation speeds.

There was no statistical difference in insular BOLD activation between caress stimulation on the arm compared to the palm, indicating a broad velocity-sensitivity without selectivity for skin type or body part. This is consistent with evidence that the PI is speed-sensitive even when simply viewing others’ stroking (Olausson et al., 2002; Morrison et al., 2011a).

The dlPFC has been implicated in both decision-making (Pochon et al., 2001; Krain et al., 2006) and reward-related processes (Leon and Shadlen, 1999; Pochon et al., 2002; Tanaka et al., 2006; Ahn et al., 2013). It is also involved in the integration of information about the outcome of previous decisions with estimations of the expected reward value of future stimuli (Barraclough et al., 2004). Here, dlPFC may have been involved in maintaining information about the relative value of the available stroking options in order to make an optimal choice (Christakou et al., 2009). Indeed, whereas neurons in macaque orbitalfrontal cortex (OFC) code the actual reward value of a reinforcer, dlPFC neurons are more related to producing the correct behavioral choice required to get a reward (Wallis and Miller, 2003; Wallis, 2007). In this perspective dlPFC reflects prospective processing by modulating behavior according to previous experiences.

This reward-choice component in our study may be especially salient for the arm. The dlPFC activation, which fell near the coordinates reported by Gordon et al. (2013) and Bennett et al. (2014) for CT-targeted touch was significantly more activated for arm than palm stroking (main effect of arm stimulation). This is consistent with prefrontal regions’ preferential activation during tactile stimulation for arm vs. palm (McGlone et al., 2012; Gordon et al., 2013) and on the general role of dlPFC in motivated decision-making, including in the tactile domain (Levy and Glimcher, 2012). Previous studies have shown that dlPFC is more activated during soft tactile stimulation on arm compared to palm (Voos et al., 2013; Bennett et al., 2014), and is temporally synchronized with amygdala activation during stroking stimulation (Gordon et al., 2013). Considering its co-activation with PI (a major target for CT projections), the dlPFC may contribute to a differential hedonic weighting of the stimulus with a dependence on skin type. dlPFC, alongside insula, may represent different hedonic weights across velocities, with PI broadly tuned to stroking velocity on both arm and palm, and the dlPFC more sensitive to skin-type-dependent factors in preference determination (Bennett et al., 2014).

### Arm and palm

The arm-palm similarities and differences that we found in this experiment may shed light on distinct and complementary cortical pathways of affective touch and their possible relationship to behavioral preferences. First of all, these findings suggest a degree of similarity between hairy skin, innervated by both CTs and Aßs (arm) and glabrous skin, innervated by Aßs (palm). The repeat-to-change ratios for both arm and palm followed an inverse U-shaped pattern, best described by a negative quadratic regressor (Löken et al., 2009; Morrison et al., 2011a).

Arm and palm stimulation and post-stimulus evaluation also activated PI and somatosensory cortices. These regions receive mixed input from both unmyelinated CT afferent pathways and discriminative tactile pathways, with predominant discriminative inputs to somatosensory cortices. Speed of stroking is a crucial feature of touch pleasantness when it comes to tactile stimulation on the hairy skin, where CT fibers are present. However, we found no significant BOLD differences for preferred speeds for arm and palm, which suggests that CT optimal stroking speeds are processed nonspecifically for both glabrous and hairy skin. So far, the CT afferent pathway has been the only afferent system observed to have a unique relationship with both stroking speed and perceived pleasantness (Löken et al., 2009; Ackerley et al., 2014), making it the prime candidate for mediating tactile processing in these specific affective terms. Yet despite having a less different speed-tuning at the afferent level, the Aß afferents that innervate both glabrous and hairy skin may also enable affective evaluation and provide an impetus to behavioral choices by virtue of their projections to interrelated cortical networks. The respective contributions of arm and palm stimulation to hedonic processing and behavior may become more apparent at a finer grain than the broad categories of “liking” and “wanting” currently allow. In real, ecological human affective touch interactions, arm and palm play different but complementary roles. The palm is an active “touch-seeking” surface used to stroke another person’s skin, and the speed of stroking should correspond to the speed that feels good to the recipient. This suggests hypotheses for future experiments involving affective evaluation not only in “strokees” but “strokers” as well (Ackerley et al., 2014; Ebisch et al., 2014). Given that there may be perceptual differences between arm and palm perception that yet have to be fully understood (McGlone et al., 2007), we suggest that the CT pathway to cortex might offer a first-pass filter from which cortical evaluative processing from glabrous skin stimulation can draw information. The activation in the caudate and dlPFC for preferred velocities indicates that slow skin stroking is not only perceived as desirable, but it might also represent a channel for the driving and regulation of behavior during affiliative touch interactions.

## Conflict of interest statement

The authors declare that the research was conducted in the absence of any commercial or financial relationships that could be construed as a potential conflict of interest.
